# Summer Hegira

**Published:** 1879-07

**Authors:** 


### SUMMER HEGIRA.

A MOST advantageous custom, and one which promotes health of body and brain, is that of citizens spending the hottest weeks of the year in the country; there can not be a doubt of its revivifying and regenerating effects, when the time is occupied in a proper manner, and the habits of eating, drinking, and exercise are dictated by a judicious reference to the ascertained laws of our being. A summering in the country will be beneficial to the body, in proportion as the whole time of daylight, from early breakfast until sundown, is spent in active pleasurable exercise in the open air; exercise which, as often as taken, should be to the extent of some little fatigue. As to young men and old, the best plan is to be afoot from morning until night, in fishing, hunting wild animals, religiously sparing the sweet birds of the wood, whose gleeful songs, as if in welcome of our arrival, ought to smite any generous heart with reproaches, for even the thought of murdering them in cold blood. To carry out this plan of health-seeking to the fullest extent, it should be arranged to go far from human habitations, and "rough it," camping out every night for weeks together, all the while dismissing business from the mind, and allowing it to feast on the beauties of nature and the goodness of our great Father, as exhibited in all that meets the eye.

As to girls and women, especially those who are burdened with family cares at home, or are weighed down with that greater load, fashionable life, the better plan is to avoid all watering-places, and away from all steamboat and railroad communication, seek in some quiet nook, in a plain, tidy farmhouse, that repose for mind and body which is so imperatively needed. A place should be sought where there are literally "no other boarders," except the members of your own family, and where there is no pretension in the household to dress and form and ceremony; where the only law is that of an honest kindness. Seek a place where there are no near neighbors; which is not immediately on any main public road; the object of all this being to enable the ladies, without wounding their self-respect, to wear the plainest, loosest clothing they possess, and to relieve them of any necessity for dressing but once in twenty-four hours, and that when they first get up in the morning, so that any moment they may wish to go out of doors, the only extra articles needed may be an old-fashioned "sun-bonnet" and a loose, light shawl; the shoes that are worn about the house should have soles nearly half an inch thick, with cork lining inside. When a lady can go out thus easily, without the necessity of changing a single garment, she will be far more apt to take a turn round the farm, to go to the spring-house, to gather eggs in the barn, to feed the chickens, to go a berrying, to visit the orchard, to pick berries for desserts, to watch the dairy-maid, to go out to the harvest-field and smell the new-mown hay, to scale fences, climb trees in the orchard, gather wild flowers, build mill-dams in the brooks, and construct artificial canals and miniature water-wheels for turning imaginary mills; to take basket on arm and botanize; or a tiny hammer, and wandering over brook and branch and hill-side and mountain-top, by the public road or the sea-side, read in every stone the geology of each locality, and much of their history through the long ages past; to row a boat, or ride a horse; to walk by the earliest dawn, or frolic by the clear moonlight of summer; all the while eating not an atom except at the three regular meals of the day; getting all the sleep possible. but only during the hours of darkness. Acting thus, few will fail of real and lasting renovation, by spending a summer in the country.

